# Survival Rate and Prognostic Factors among Iranian Breast Cancer Patients

**Published:** 2020-02

**Authors:** Mojtaba MESHKAT, Ahmad Reza BAGHESTANI, Farid ZAYERI, Maryam KHAYAMZADEH, Mohammad Esmaeil AKBARI

**Affiliations:** 1. Department of Biostatistics, School of Paramedicine, Shahid Beheshti University of Medical Sciences, Tehran, Iran; 2. Physiotherapy Research Center, Department of Biostatistics, Faculty of Paramedical Sciences, Shahid Beheshti University of Medical Sciences, Tehran, Iran; 3. Cancer Research Center, Shahid Beheshti University of Medical Sciences, Tehran, Iran

**Keywords:** Cure fraction models, Breast cancer, Survival probability

## Abstract

**Background:**

Survival time is one of the indicators used for evaluation of the quality of care in different types of malignancies, including breast cancer. The present study aimed to estimate the survival rate of breast cancer and its related factors among Iranian patients.

**Methods:**

Overall, 3148 cases of breast cancer who referred to the Cancer Research Center in Shahid Beheshti University of Medical Sciences, Tehran, Iran during 1994–2017 participated in this longitudinal study. Survival estimates were calculated using the Kaplan-Meier method and the Bayesian generalized Birnbaum–Saunders model with cure rate from geometric distribution. Clinical, pathological, and biological variables as potential prognostic factors were entered in univariate and multivariate analyses.

In order to identify the significant prognostic factors, 95% highest posterior density (HPD) intervals were used.

**Results:**

The overall 1, 5, 10, 15, 20 and 25-year survival rate were 95%, 75%, 60%, 47%, 46% and 46%, respectively. A significant relation was observed between survival time and the variables such as age, size of tumor, number of lymph nodes, stage, histological grade, estrogen receptor, progesterone receptor, and lymphovascular invasion.

**Conclusion:**

The findings of this study might help the health managers to plan long-term programs considering regional determinants, public education, and screening for early detection of breast cancer cases which can eventually influence the overall survival rate of these patients.

## Introduction

Breast cancer is the most common non-skin cancer among women and is the second cause of mortality due to cancer in the world (1–3). Based on the latest statistics of Globocan in 2012, the breast cancer incidence was 1671 thousand cases in the world while 39% of the diagnosed cases were in Asian countries (4). About 249260 new cases of breast cancer were diagnosed in America in 2016 among which 40890 cases (16%) of mortality were reported (5). However, the mortality rate, due to this cancer, varies in different countries (1, 4–6). Due to the early diagnosis and improvement of treatment methods, the number of mortality has reduced in some western countries (2) while the mortality rate is more among the developing countries due to a low level of awareness and late diagnosis (4, 7, 8). The survival rate was reported 73% in developed countries and 53% in developing countries (4, 7).

Breast cancer is the fifth cause of mortality in Iranian women (8–12). Based on the statistics of cancer in Iran, its incidence was reported 7582 cases (23% of total cancers in women) in 2009 and its age was, unfortunately, decreasing (9). The 1-, 3-, 5-, and 10-year survival rate of breast cancer in Iran were 95.8%, 82.5%, 69.5%, and 58.1%, respectively (13).

The most important factors influencing the survival of these patients included genetic changes, hormonal effects, (1, 6, 14) and environmental variables (2, 3). In general, all these factors can affect the growth, colonization, emission, and longer survival of the suffered person directly or indirectly to create a specific texture of breast cancer (15, 16). Thus, based on the world health organization report, more than 30% of cancer mortalities have been reduced by controlling the risk factors.

There are different treatments for breast cancer based on the disease stage, histology characteristics, biomarkers level, biologic subgroup, and general status of the patient (14). Surgery is regarded as the most important treatment. Other methods can be used for the remaining and non-identifiable parts of the disease (6). There are two surgery methods of MRM (modified radical mastectomy) or total mastectomy and BCS (breast-conserving surgery) (6, 14).

Despite the accurate statistics for the breast cancer survival rate, its effective factors have not completely recognized yet. In addition, the reported cases on this disease vary in different areas (9). Based on the increase of incidence of breast cancer since 1990 in most countries (1) and its increasing growth, especially at lower ages in Iran (8) and its high treatment costs, the broad controlling programs are necessary to improve, prevent, and recognize the factors affecting the survival and use the healthcare budget optimally (8, 17, 18).

Survival analysis models are a set of statistical methods for the data in which the intended response variable is the time until the occurrence of an event (19). The high rate of censorship in survival data created a bias in maximum likelihood in a variety of standard survival models (20). In survival studies, the risk function for every person may depend on a set of risk factors or explanatory variables. All these risk factors are called “Individual heterogeneity or frailty” (21). A specific state of binary frailty was dividing the population to the people at risk and those who were not subject to risk. In such models, the people who have never experienced the desired event until the end of the study were called “cured” (22). In fact, in these models with the increase in the duration of the study, the probability of occurrence an event does not go towards one (23). The advantage of using cured models than conventional survival models was that, in addition to studying the factors affecting the survival function, the cure fraction and the factors affecting it can be analyzed separately and more accurate estimates of the amount of effect of the independent variables on survival time (24). In the present study, the extended Birnbaum–Saunders distribution was used for the survival time of patients. This model was introduced for the first time by Volodin and Kazan in 2000 (25). The generalized Birnbaum–Saunders distribution had more flexibility due to the inclusion of a new parameter to the model and led to the improvement of the model (26–28). Thus, the present study aimed at investigating the survival rate and prognostic factors of Iranian breast cancer patients.

## Materials and Methods

### Study design

In this study, 3184 patients with breast cancer referring to cancer research center of Shahid Beheshti University of Medical Sciences, Tehran, Iran during 1994–2017 were studied. All pathology of breast cancer was confirmed by a pathologist. The male patients with breast cancer and those with incomplete information in files were excluded from the study.

In this longitudinal study (29), the analysis of data extracted from the cancer research center of Shahid Beheshti University was conducted in Tajrish Shohada Hospital.

This project was studied and confirmed by the research committee of Cancer Research Center and the Ethics Committee of Shahid Beheshti University of Medical Sciences (IR.SBMU.RETECH.REC.1395.750). All information related to the patients was considered confidential.

### Variables of the study

Dependent variable included the mortality rate of patients due to breast cancer (event) and the survival time (year). Tumor size was categorized into 3 groups: less than 2 as T1, 2–5 as T2 and more than 5 cm as T3+; tumor grade was categorized into 3 degrees of I, II and III+; cancer stage was categorized as Well, Moderately and Poorly; number of metastatic lymph nodes was categorized to 3 sizes (1–2 as N0, 3–5 as N2 and more than 5 as N3+); type of surgery(MRM, BCS), Estrogen Receptor (ER, negative or positive), Progesterone Receptor (PR, negative or positive) and age at the diagnosis time categorized to 3 age groups (less than 40, 40 to 60 and older than 60 yr) were regarded as independent variables. In addition, the patients who did not die during the study were considered as censor (83%).

### Statistical method

The data were presented in number (%), Mean ± SD and we used Kaplan-Meier method to analyze descriptive data. The Bayesian generalized Birnbaum–Saunders distribution with cure fraction and geometric distribution was used for the first time. Markov chain Monte Carlo (MCMC) methods were used for estimating the parameters. The software used in this analysis was SAS, University Edition (SAS Institute, Cary, NC, USA). In order to identify the significant prognostic factors, 95% highest posterior density (HPD) intervals were used.

## Results

The results are reported in two sections: descriptive and analytical analysis of the cure Model.

### Descriptive analysis

In this longitudinal study, 3184 breast cancer women with a mean age of 49±12 yr were studied (Table 1).

**Table 1:** Clinical, pathological, and biological characteristics in patients with breast cancer

***Variable***		***n (%)***	***Death (%)***	***Estimated Survival time (Years)***		***95% Confidence Interval***	
				**Mean**	**Std. Error**		
Age at diagnosis	Under 40 yrs.	670(21.0)	116(17.3)	16.0	0.76	14.5	17.5
	40 to 60 yrs	1926(60.5)	319(16.6)	14.6	0.49	13.7	15.6
	Over 60 yrs	588(18.5)	101(17.2)	13.0	0.92	11.2	14.8
Histologic grade	Well	391(12.3)	19(4.9)	21.4	0.86	19.8	23.1
	Moderately	1680(52.8)	272(16.2)	15.2	0.51	14.2	16.2
	Poorly	1113(35.0)	245(22.0)	11.4	0.63	10.2	12.7
Stage	I	663(20.8)	24(3.6)	21.3	0.81	19.7	22.9
	II	1467(46.1)	210(14.3)	16.2	0.58	15.0	17.3
	III+ (III & IV)	1054(33.1)	302(28.7)	10.0	0.54	8.9	11.0
Lymph node status	N0	1479(46.5)	96(6.5)	19.7	0.56	18.6	20.8
	N1	1389(43.6)	337(24.3)	11.9	0.57	10.8	13.0
	N2+(N2 & N3)	316(9.9)	103(32.6)	7.5	0.66	6.2	8.8
Tumor size	T1	1009(31.7)	66(6.5)	19.0	0.75	17.6	20.5
	T2	1707(53.6)	308(18)	14.5	0.54	13.5	15.6
	T3+(T3 & T4)	468(14.7)	162(34.6)	9.5	0.75	11.0	5.3
Estrogen receptor	Negative (−)	2278(71.5)	428(18.8)	14.5	0.45	13.6	15.3
	Positive (+)	906(28.5)	108(11.9)	16.3	0.82	14.7	17.9
Progesterone receptor	Negative(−)	2126(66.8)	409(19.2)	14.5	0.46	13.6	15.4
	Positive (+)	1058(33.2)	127(12.0)	15.5	0.72	14.0	16.9
Lymphovascular invasion	Negative(−)	1817(57.1)	159(8.8)	18.7	0.48	17.8	19.7
	Positive (+)	1367(42.9)	377(27.6)	7.7	0.33	7.1	8.3
Type of surgery	BCS	1997(62.7)	250(12.5)	16.2	0.67	14.9	17.5
	MRM	1187(37.3)	286(24.1)	14.2	0.50	13.3	15.2

**Fig. 1:**
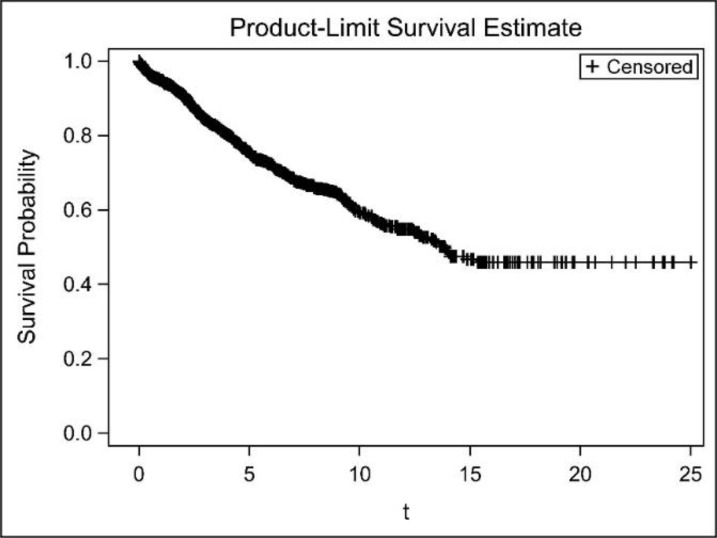
Kaplan–Meier estimate of the surviving function in breast cancer data

The average mortality rate was about 17% in different age groups of breast cancer patients in which, their survival rate reduced with an increase in mean age. An increase in grade, stage, number of positive lymph nodes, and size of tumor resulted in increasing the mortality rate to seven times than women with less mortality rate and reducing their survival rate to 12 years. In addition, the mortality rate of patients with attack on lymph nodes was three times higher than patients without attack and their survival rate was 11 years less than the women without attack on lymph nodes. The mortality rate of ER (+) patients was less than ER (−) ones and the same trend was also observed for PR(+) patients. The average survival time of the patients under BCS surgery was more than the patients under MRM surgery and the mortality rate of the patients under BCS was almost half of the MRM patients. Figure 1 illustrates the Kaplan–Meier survival diagram. As shown, 1, 5, 15, 20, and 25- year survival probabilities of the patients were 95%, 75%, 60%, 47%, 46%, and 46% respectively. The women who survived more than 15 years after their cancer had a survival function of straight line with no reduction in survival probability, called the cured women. Thus, using the cure models was an appropriate option for such data (Fig. 1).

### Analytical Analysis

Table 2 indicates the fitness results of the cured survival model. The patients less than 40 yr old had significantly more probability of cure than the 60-year-old subjects (HPD: 0.117, 0.808).

**Table 2:** Estimation based on non-mixture cure rate model

***Variable***		***β***	***sd***	***Percentile***			***Exp(β)***	***95% HPD Interval***	
				***25***	***50***	***75***			
Age at diagnosis	Under 40 yrs.	0.461	0.179	0.338	0.462	0.584	1.586^*^	0.117	0.808
	40 to 60 yrs.	0.283	0.149	0.182	0.283	0.386	1.328^*^	0.002	0.577
	Over 60 yrs.	REF							
	No	REF							
Type of surgery	BCS	−0.034	0.113	−0.112	−0.031	0.043	0.967	−0.245	0.190
	MRM	REF							
Tumor size	T1	1.325	0.190	1.190	1.325	1.454	3.761^*^	0.959	1.688
	T2	0.523	0.137	0.429	0.523	0.616	1.687^*^	0.244	0.778
	T3+	REF							
Lymph node status	N0	1.011	0.210	0.868	1.013	1.155	2.747^*^	0.623	1.435
	N1	0.081	0.156	−0.026	0.085	0.188	1.085	−0.212	0.388
	N2+	REF							
Stage	I	0.618	0.265	0.434	0.611	0.793	1.854^*^	0.121	1.158
	II	0.044	0.132	−0.047	0.040	0.131	1.045	−0.211	0.304
	III+	REF							
Histologic grade	Well	1.097	0.269	0.915	1.095	1.272	2.995^*^	0.599	1.639
	Moderately	0.203	0.113	0.128	0.204	0.279	1.225	−0.012	0.434
	Poorly	REF							
Lymphovascular invasion	Positive(+)	−1.139	0.122	−1.221	−1.143	−1.056	0.320^*^	−1.373	−0.900
	Negative(−)	REF							
Estrogen receptor	Positive(+)	0.440	0.163	0.330	0.442	0.551	1.553^*^	0.137	0.771
	Negative(−)	REF							
Progesterone receptor	Positive(+)	0.475	0.158	0.374	0.473	0.582	1.608^*^	0.165	0.781
	Negative(−)	REF							

* ) significant based on 95% HPD interval.

In addition, the people aged between 40–60 yr had 32.8% probability of cure which was significantly more than the people above 60 yr old(HPD: 0.002, 0.577).

**Fig. 2:**
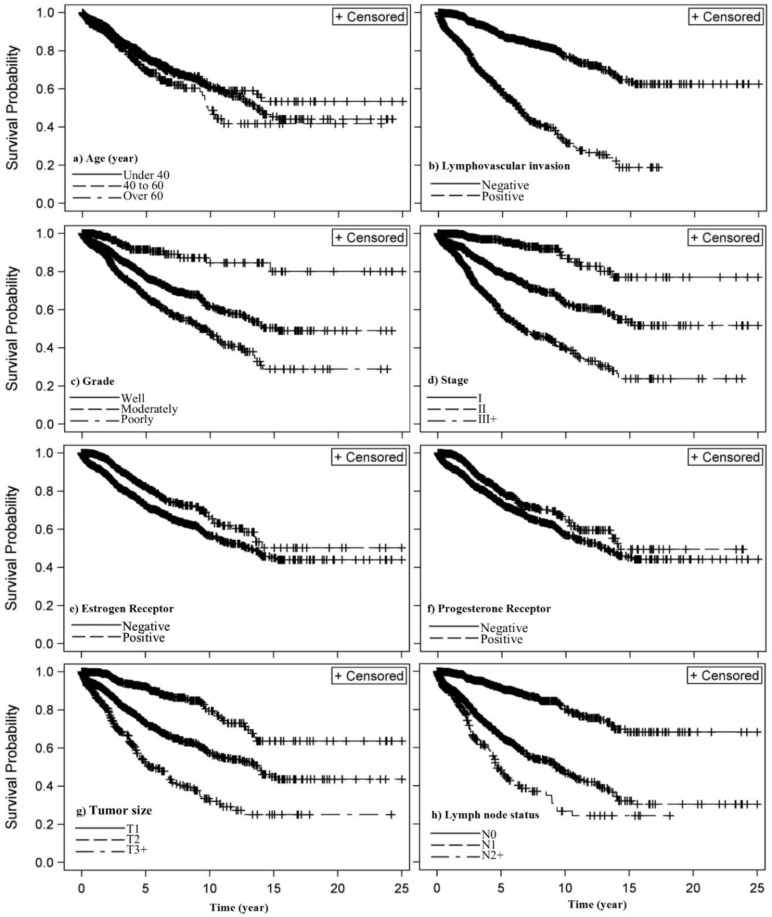
Kaplan–Meier estimate of the surviving function for effective significant variables of breast cancer

Cure rate in the women with tumor size of T1 and T2 was 3.76 (HPD: 0.959, 1.688) and 1.68 (HPD: 0.244, 0.788) times higher than the T3+ women, respectively and cure rate in the women with positive lymph nodes N0 was 2.75 (HPD: 0.623, 1.435) times higher than the N2+ women which were statistically significant. Although the cure rate N1 was 8% more than N2+ women it was not statistically significant. Cure rate in women with stage 1 was 85% (HPD: 0.121, 1.158) significantly more than the cure rate in stage 3. Although cure rate in stage 2 patients is more than the rate in stage 3, the difference was not statistically significant. In women with grade 1(well), the cure rate was 2.99 (HPD: 0.599, 1.639) higher than the women with grade 3 (poorly) which was statistically significant. Although cure rate in grade 2 (moderately) was more than that of grade 3, the difference was not statistically significant. The cure rate of the women without Lymphovascular invasion was 3.12 (**1/.320=3.125**) (HPD: −1.373, −0.900) times higher than that of the women with Lymphovascular invasion which was statistically significant. The cure rate of the women with estrogen and progesterone receptor was respectively 55.3% (HPD: 0.137, 0.771) and 60.8% (HPD: 0.165, 0.781) more than woman without receptors which was statistically significant. In addition, there was no significant relationship between types of surgery. Figure 2 illustrates the Kaplan-Meier function of the variables related to the patients’ survival based on the results in Table 2.

## Discussion

Overall 1, 5, 10, 15, 20 and 25-year survival rate of breast cancer in Iran were 95%, 75%, 60%, 47%, 46% and 46%, respectively. A significant relation between survival time and the variables such as age, size of tumor, number of lymph nodes, stage, histological grade, estrogen receptor, progesterone receptor, and lymphovascular invasion was observed.

The most important limitation of this study was an incomplete recording of the data resulted in the elimination of 800 cases. It would be of interest to recommend medical centers to record more accurately using electronic software for data collection.

Compared to the previous studies conducted in Iran, our study showed an increase in the 5-year survival rate due to an increase in awareness or improvement of treatments (11, 12, 30, 31).

The high rate of 5-year survival in Iran indicated the optimal follow-up and appropriate awareness of such a disease. However, due to an increase in breast cancer incidence in Iran and lower survival rate of 8%, compared to the statistics in America and Europe, a more comprehensive planning is critical in this field (4, 32).

In a series of studies in Iran, the mean age of cancer people was 48.4, 47.0 and 46.8, respectively while in our study the mean age equaled to 49.0 (10, 17, 31, 33). This result is in line with the study of that indicated an increase in patients’ mean age, which can due to an increase in awareness or more effective treatment of patients. In the present study, the 5-year survival probability among the women below 40 yr, 41 to 60, and more than 60 yr were 91, 80, and 69 yr, respectively. Furthermore, the present study indicated that the cure probability of women less than 40 yr, and between 41 to 60 yr were 59.2% and 33.3%, respectively, which significantly more than the women with over 60 yr old. Although the 5-year survival rate for different age groups was not considered in any study, another study showed that the survival rate in patients less than 40 yr with an age range of 50–60 was more than the range of 60 yr (31). Age was not observed as an independent factor for survival, due to the lack of an obvious difference of other factors among different age groups with insufficient sample size in their study (34).

Based on the results of this study, the patients’ death increased up to seven times with an increase in the stage while the survival duration reduced to 24%. In addition, based on the results of fitting the model, the cure rate of women with stage 1 was 85% more than the women with stage 3 which was statistically significant. However, the cure rate in women with stage 2 was more than stage 3 although the difference was not statistically significant. Similar to the present study, a significant relationship was confirmed between stage and survival, in a series of studies (10, 11, 18, 30, 31). According to our results, the cure rate increased from 68% to 3.76% times with a decrease in tumor size. The cure rate in T1 patients was about three times more than the T3+ patients while this rate in T2 patients was 68% more than the T3+ patients. Another study showed a significant relationship between survival and tumor size. In the study of Mahood et al., no significant relationship was observed between the tumor size and total survival of patients (35). The results of other studies proved the reverse effect of tumor size on survival (10, 31).

The present finding also supports other studies, which concluded that the involvement of lymph node is a very important factor for the cancer patients’ survival (36, 37). In addition, the mortality rate of the patients with lymphovascular invasion was three times more than that of other women and their survival rate was 36.3% less than the women without lymphovascular invasion. Cure rate in women without lymphovascular invasion was 3.12 times more than the patients with lymphovascular invasion. Cure rate in women with no lymph node was 2.75 times more than the N2+ women which were statistically significant. However, despite the more mortality rate of N2+ women in comparison with N1 women, no significant difference was observed between N1 and N2+ related to the difference in the effect of other items of TNM system such as the size of tumor and distant metastasis in N1 group. In line with the results of the present study, the patients with the involvement of more than 10 lymph nodes had 12 months less survival and 24% more mortality (31). In another study, a significant relationship was shown between survival and lymph node involvement (10). The results of some other studies were congruent with the results of the present study, Peng et al. indicated a decrease in survival rate with an increase in lymph node involvement (38), another study showed a significant relationship between auxiliary lymph node involvement and prognosis except in the age range of 35–39 yr (34), Khodabakhshi (11) showed the number of lymph node involvement as a factor affecting the survival rate, which confirmed by other studies (39).

The results of the present study indicated that the cure rate reduced to three times with an increase in tumor grade. Moreover, the mortality rate increased to seven times and their survival reduced to 24.5%. In women with grade 1, the cure rate was 2.99 times higher than the women with grade 3, which was statistically significant. Although the cure rate of grade 2 was more than that of grade 3, the difference was not statistically significant. Similar to the results of this study, there is a significant relationship between the disease grade and survival of the person (10, 31). Furthermore, the survival rate reduced and mortality rate increased with an increase in grade (11).

We have shown that the mortality rate in the patients with positive estrogen and progesterone receptors were less than negative receptors. The cure rate in these patients was more than the negative hormone receptors which were statistically significant. In line with the results of the present study, the presence of positive Estrogen and Progesterone increased cure probability to 8% and 16% (31). In a series of studies (11, 31, 40, 41), no significant relationship was found between survival time and progesterone receptors.

In the present study, the mortality rate in patients under MRM was twice more than that of the patients under BCS. Besides, the cure rate of patients under BCS and MRM was almost similar and no significant difference was observed. The patients under MRM had survival more than 20 months and their mortality percent in the patients under MRM was more than BCS surgery (31). However, the patients under BCS had more survival than those under MRM (7). The reason for such contradictory data could be for the method of selection of candidates for MRM who were not at higher stages of the disease and could not be compared to those with lower levels of disease and lymphovascular invasion for BCS.

## Conclusion

In general, clinical and pathological factors play a significant role in the survival rate of breast cancer patients. The results of this study will help health managers to provide long-term plans to predict patients’ status and accordingly therapeutic policies. In addition, the need for greater awareness of women for screening and early detection of breast cancer is obvious due to the apparent effect observed on the survival of patients.

## Ethical considerations

Ethical issues (Including plagiarism, informed consent, misconduct, data fabrication and/or falsification, double publication and/or submission, redundancy, etc.) have been completely observed by the authors.
